# Fabrication and Tribological Performance of Zr-Coated Carbide against 40Cr Hardened Steel

**DOI:** 10.3390/ma11071248

**Published:** 2018-07-20

**Authors:** Wenlong Song, Zixiang Xia, Shoujun Wang, Xuan Zhang

**Affiliations:** 1 Department of Mechanical Engineering, Jining University, Qufu 273155, China; shoujun0531@163.com (S.W.); 13105474878@163.com (X.Z.); 2 Department of Material Science & Engineering, Shandong University, Jinan 250061, China

**Keywords:** cemented carbide, Zr coating, multi-arc ion plating, tribological performance

## Abstract

In order to enhance the tribological performance of YT14 carbide, pure Zr coating was deposited on the substrate surface using a multi-arc ion plating method. The surface topography, adhesion strength, thickness, and micro-hardness of the Zr coating were tested. Dry sliding friction experiments against a 40Cr hardened steel ring were conducted with Zr-coated carbides and traditional ones. The average coefficients of friction were measured and compared. The wear characteristics of the samples were examined by scanning electron microscope (SEM) and energy dispersive X-ray analysis (EDX). The test results indicated that the Zr coating deposited on the carbide surface exhibited excellent adhesive strength and lower hardness. The average friction coefficient of Zr coated carbide decreased by 20%–30% in comparison with that of the uncoated one. The Zr coated carbide could reduce the adhesive wear compared with the uncoated one, and the main tribological degradation mechanisms of the coating were abrasive wear, coating flaking and delamination.

## 1. Introduction

Cemented carbide has been widely applied in engineering productions due to the advantages of high strength, and excellent anti-wear properties [1]. However, in high-speed cutting and hard cutting, the carbide exhibits higher friction coefficient, lower wear resistance, and a short lifetime [2]. Coating on the carbide surface is an economical and effective technique, which includes hard coatings and soft coatings, to improve the tribological performance and wear resistance, which include hard coatings and soft coatings.

With higher hardness, better excellent abrasion resistance, and high-temperature stability, hard coatings are applied to improve wear resistance and enhance the tribological performance of substrate materials. First generation hard coatings were simple binary composite materials such as TiN, ZrN, and CrN [3,4,5,6]. The successful application of the early generation hard coatings resulted in the rapid development of the next generation physical vapour deposition (PVD) multi-component composite coatings and their combinations as multilayers, which provide higher hardness and better wear resistance. The multi-component composite coatings, such as TiAlN, AlCrN, TiSiN, TiAlSiN, and CrSiCN, have attracted a lot of research because of the solid solution strengthening [7,8,9,10,11,12,13,14,15,16,17]. The multi-component composite coatings exhibit much higher hardness and better tribological properties than binary coatings, and the addition of metal during deposition process produces much more stable substitution solid solutions. 

Soft coatings of substrate are considered to be a kind of effectual method for improving friction performance by forming a layer of lubrication film, and they have been found to significantly improve tribological characteristics due to the lubricating effect, such as tungsten disulfide (WS_2_) [18,19,20,21], molybdenum disulfide (MoS_2_) [22,23,24,25], etc. These sulfides are well-known as dispersants in cutting coolant, polished coatings, and coatings prepared by physical vapor deposition (PVD) technique, PVD being a promising method of fabrication of sulfide coatings. To further enhance the wear resistance and service life of sulfide coatings, the combination of sulfides and metals such as Ti, Cr, or Zr have been found to significantly promote mechanical properties and tribological performance [26,27,28,29,30,31,32,33,34,35], and have been widely used in industrial productions.

However, the present research mainly focuses on hard coatings and soft coatings with a combination of metals (e.g., Ti, Cr, Zr). Information about the properties of pure metal-coated carbide inserts is still very scarce [36,37]. These need to be further studied in order to expand the application range.

In the present work, pure Zr coating was deposited onto the surface of YT14 (WC + 14%TiC + 6%Co) carbide using multi-arc ion plating technology. The surface morphology, adhesion strength, thickness, and micro-hardness of the Zr coating were evaluated. Dry sliding friction experiments of the Zr coated carbides against 40Cr hardened steel ring were conducted by employing a ring-block method. The friction characteristics of the Zr coated carbides against hardened steel were investigated.

## 2. Experimental Details

### 2.1. Preparation of Pure Zr Coating

YT14 carbide (WC + 14%TiC + 6%Co) was employed as the coated substrate material. Composition and principal characteristics of YT14 carbide are displayed in Table 1. The surface of the sample was burnished to a mirror finish, then washed ultrasonically in acetone for 20 min, and lastly dried in a prevacuum dryer for about 30 min. The pure Zr coating was deposited by multi arc ion plating method using two Zr targets (99.99%). The coating deposition parameters are shown in Table 2.

The adhesion strength between the Zr coating and YT14 substrate and thickness was measured with the MT-4000 Material Surface Properties Tester, by scratching the Zr coating′s surface with a diamond tip with a radius of 200 μm. The adhesion strength tests were implemented with an applied force of 80 N, with the force increasing at a rate of 80 N/min and a sliding distance of 10 mm. The measurement of coating thickness were performed with a sliding distance of 6 mm and a testing time of 60 S. The surface micro-hardness of the coating was measured on the MH-6 micro-hardness meter with an applied load of 0.2 N. The surface topography of the coating was obtained with a white light interferometer.

### 2.2. Sliding Friction Tests

**Figure 1:**
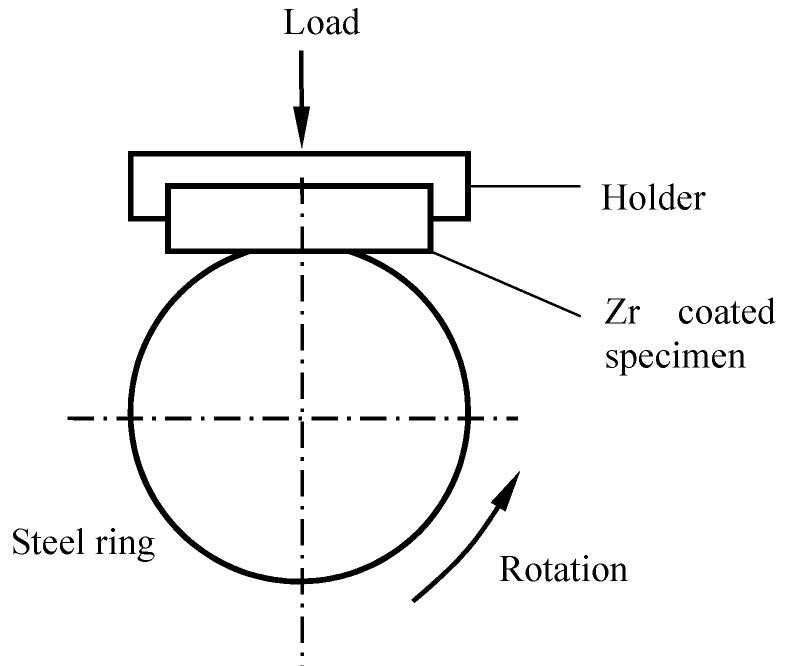
Schematic diagram of the wear testing with ring-block tribometer.

Sliding friction tests were carried out on a MRH-3 high-speed ring-block tribometer. The schematic of the ring-block tester is indicated in Figure 1. The upper block (15 mm × 15 mm × 4.5 mm) was Zr-coated carbide. The lower ring sample (*Φ*50 × *Φ*35 × 15 mm) was a 40Cr hardened steel ring with a surface hardness of HRC 45–48. The Zr-coated sample was mounted in a holder, while the 40Cr ring was rotated at a speed of 100–400 rpm. The applied normal load was in the range of 20–50 N, and the sliding time was 5 min. The average friction coefficient was the ratio of tangential force to the normal force. 

All the tests were carried out three times for average values. To explore the friction performance and properties of the pure Zr coating, measurements were also executed via SEM (INCA Penta FETXS, Oxford Instruments, Abingdon, UK) and EDX (D8 ADVANCE, Bruker, Karlsruhe, Germany).

## 3. Results and Discussion

### 3.1. Properties of the Zr Coating

**Figure 2:**
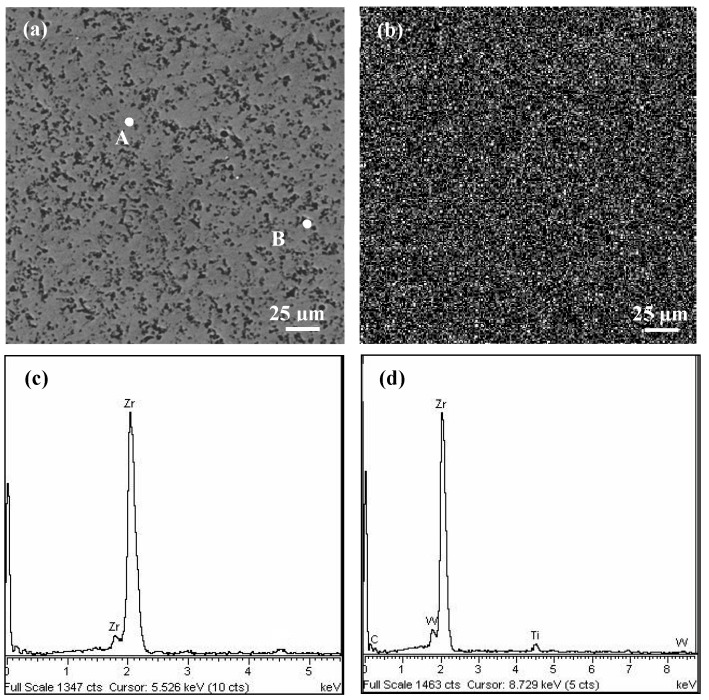
Scanning electron microscopy (SEM) micrograph and energy dispersive X-ray analysis (EDX) spectrum element analysis of the Zr coating: (**a**) surface micrographs; (**b**) Zr element distribution in (**a**); (**c**) and (**d**) corresponding composition analysis of point A and B in (**a**).

Figure 2a exhibits surface micrograph of the pure Zr coating. The EDX spectrum map of Zr element distribution on the coating surface is illustrated in Figure 2b. Figure 2c,d exhibits the corresponding EDX spectrum composition analyses of point A and B in Figure 2a, and the element content of the two points are shown in Table 3. It was demonstrated that the Zr element existed in the surface coating, and the element was relatively evenly distributed throughout the structure of the coating.

**Figure 3:**
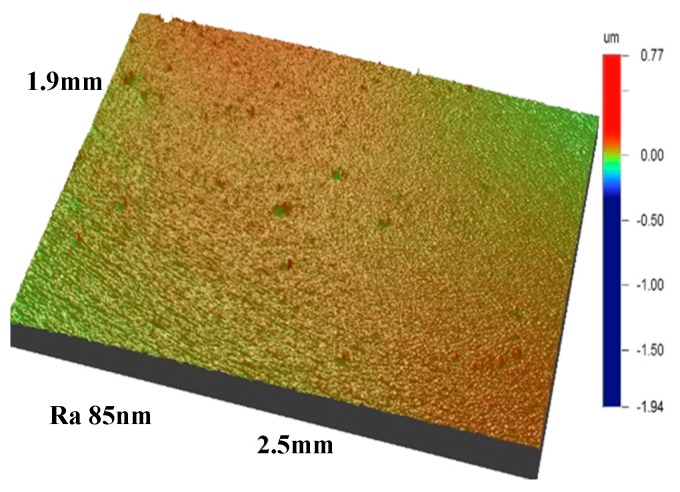
The surface topography of Zr coating detected by a white light interferometer.

Figure 3 indicates the topography of the Zr coating surface tested via a white-light interferometer. As indicated, the surface of the Zr coating was quite smooth and compact; and the value of surface roughness reached about Ra 85 ± 5 nm.

**Figure 4:**
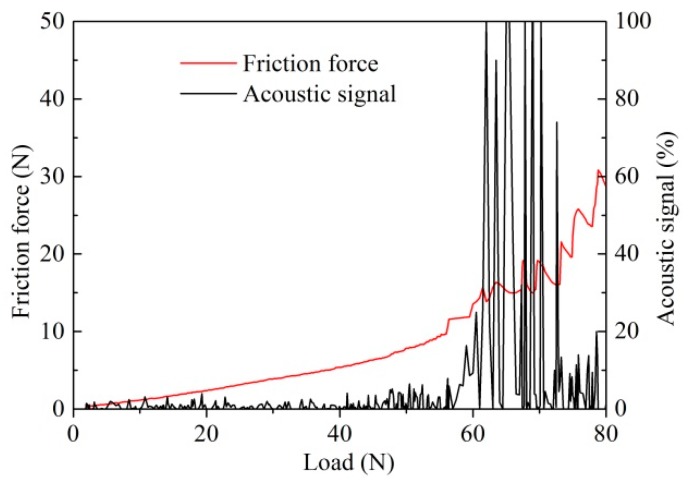
Adhesion strength of the Zr coating.

The adhesion strength was determined as the signal variations of friction force and acoustics due to the spalling of coating. The curves of friction force and acoustic signals in scratching tests are plotted in Figure 4. At the beginning of the scratching test, the frictional force curve was steady and smooth, and the acoustic signal was so small that it could be ignored. As coating failure began and the coating scraped off gradually, the fluctuations of friction force and acoustic signal increased significantly. From Figure 4, the adhesion strength of the Zr coating was considered to be about 60 ± 5 N.

**Figure 5:**
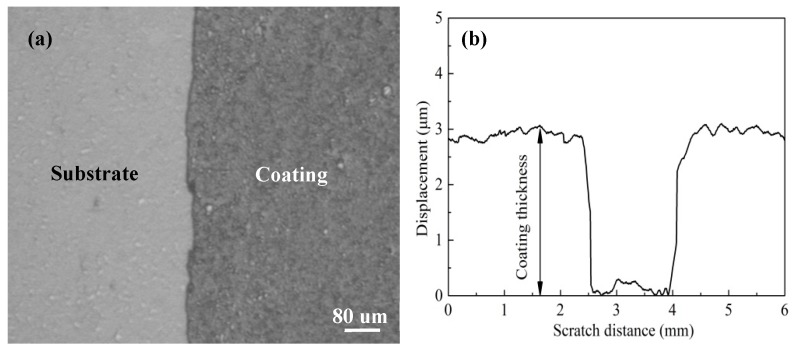
Micrograph of coating-substrate interface (**a**) and thickness curve by scratch test (**b**).

Coating thickness was obtained through measuring the difference in height between the coated and uncoated carbide sample as indicated in Figure 5a. Figure 5b presents the thickness curve of scratching test with the MT-4000 Tester; and the coating thickness was determined to be about 3.0 ± 0.1 μm.

The surface micro-hardness, adhesion strength, thickness, and surface roughness of the Zr coating are exhibited in Table 4. It shows that the coating hardness was just 12.0 ± 0.5 GPa, which was reduced by 22% in comparison with that of the YT14 substrate (15.4 ± 0.5 GPa).

### 3.2. Tribological Behaviors of Zr Coating

**Figure 6:**
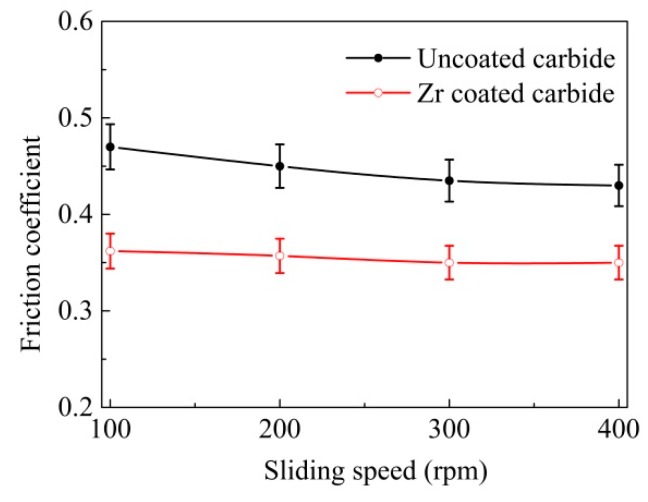
Friction coefficient as a function of sliding speed of the coated and uncoated carbides (normal load 20 N, sliding time 5 min).

**Figure 7:**
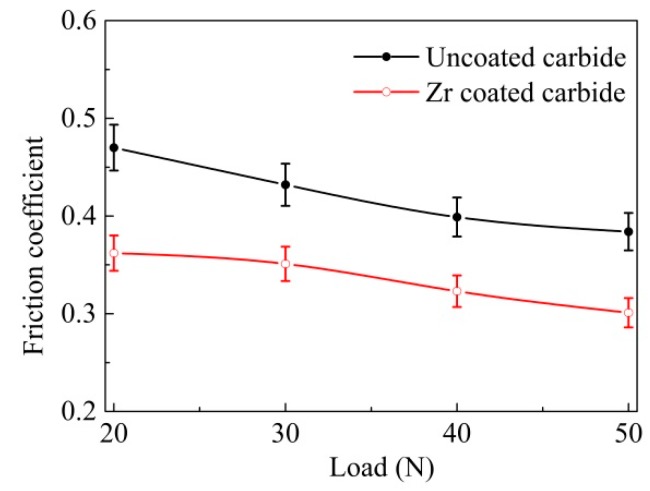
Friction coefficient as a function of applied load of the coated and uncoated carbides (sliding speed 200 rpm, sliding time 5 min).

The average friction coefficients of the Zr-coated carbide and the uncoated YT14, against 40Cr hardened steel under different sliding speeds and loads, are exhibited in Figure 6 and Figure 7 respectively. It was evident that the friction coefficients of uncoated carbide were 20%–30% higher than those of the Zr -coated one under the same experimental conditions. In Figure 6, the friction coefficients of Zr-coated sample stabilized at about 0.35–0.36, and the sliding speed changed from 100 to 400 rpm, while the friction coefficients of YT14 reached 0.43–0.47. As can be seen in Figure 7, it was found that the coefficients of friction were reduced along with the increasing load. As the applied load increased from 20 to 50 N, the friction coefficients of the coated sample were decreased from 0.37 to 0.30, while those of YT14 were reduced from 0.47 to 0.38. The test results indicated that the Zr coating can reduce the friction coefficient of the carbide under the similar test conditions, compared to the ZrN coating with the friction coefficient of 0.40–0.50 [6].

The average friction coefficient of friction pairs under elasticity loaded conditions can be expressed with the formula as follows [38]:

(1) $$\mu =\tan\beta =\frac{F_{f}}{P}=\frac{{\bar{\tau }}_{c}A_{r}}{\sigma_{b}A_{r}}=\frac{{\bar{\tau }}_{c}}{\sigma_{b}}$$

where *β* is the friction angle, *F*_f_ is frictional force, *P* is the applied load, *A*_r_ is actual contact area, *σ*_b_ is the compressive yield limit of the substrate materials, and ${\bar{\tau }}_{c}$ is the average shear stress of sample surface.

The compressive yield limit of the YT14 carbide substrate remains basically unchanged [38]. Then the equation indicates that the decreased average shear stress of the carbide surface contributes to reduce the friction coefficient. Because the shear stress of the pure Zr coating is lower than that of the carbide substrate, Zr-coated carbide is propitious to reducing the average friction coefficient, and this corresponds with the variation curve of friction coefficient illustrated in Figure 6 and Figure 7.

### 3.3. Wear Surface Studies

**Figure 8:**
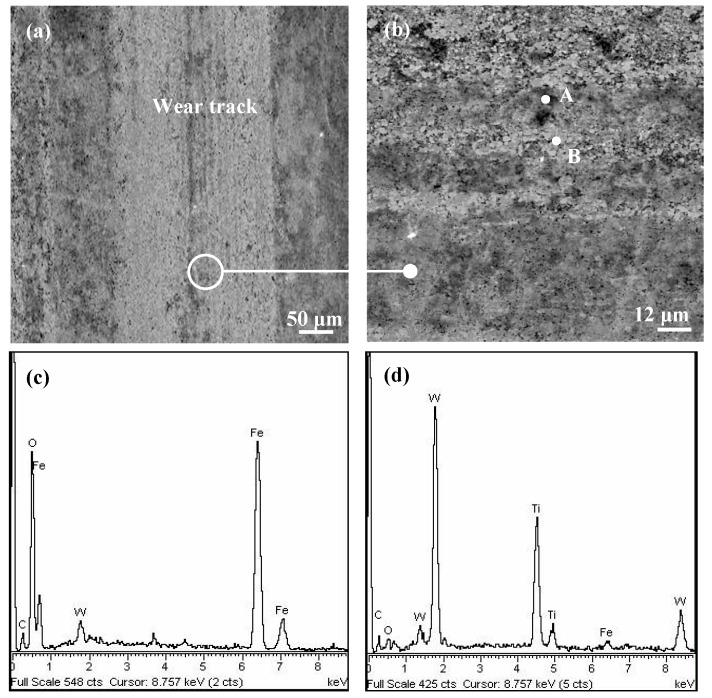
SEM micrographs and EDX spectrum analysis of the worn surface of uncoated YT15 carbide after 5 min sliding operation at a speed of 200 rpm and a load of 20 N: (**a**) worn face; (**b**) enlarged SEM corresponding to (**a**); (**c**) and (**d**) EDX spectrum analysis of points A and B in (**b**).

To better study the friction and wear features of the tested carbides, SEM and EDX were utilized to investigate the worn micrograph and element composition on the worn zone. Figure 8 indicates the surface micrographs and element composition analysis on the worn track of the uncoated carbide after 5 min sliding duration with the applied load of 20 N and slide speed of 200 rpm. There existed obvious abrasive wear on the wear surface (Figure 8a), and adhering materials could be observed on the worn track (Figure 8b). The corresponding element composition analysis (Figure 8c,d) confirmed that there existed Fe and O elements in addition to the elements of the carbide substrate. These additional elements were considered to be transferred from the 40Cr ring, owing to the severe friction between the uncoated carbide and steel sliding pair.

**Figure 9:**
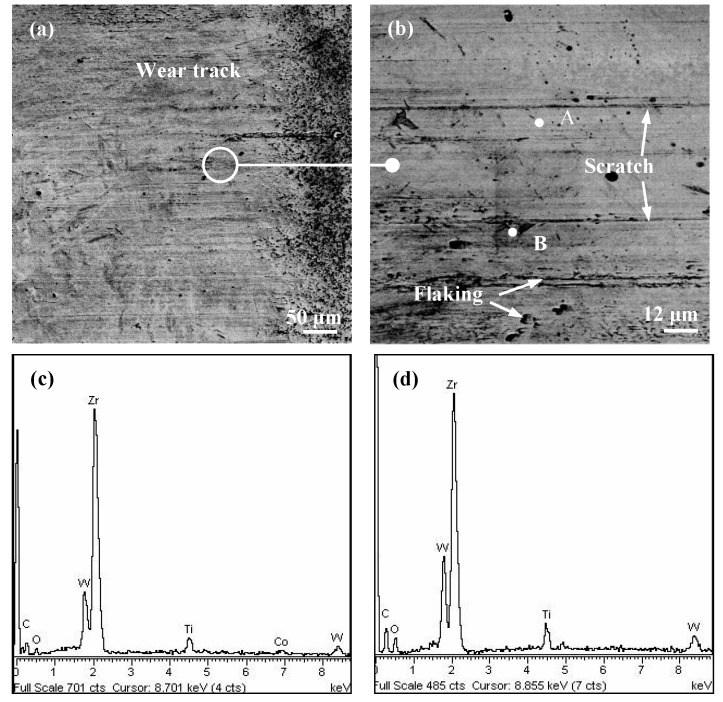
SEM micrographs and EDX spectrum of the worn surface of Zr coating after 5 min sliding operation at a speed of 200 rpm and a load of 20 N: (**a**) worn face; (**b**) enlarged SEM corresponding to (**a**); (**c**) and (**d**) EDX spectrum analysis of point A and B in (**b**).

Figure 9 shows the surface topographies and composition analysis on the worn track of the Zr-coated sample. Significant abrasive wear was found with the distinctive features of mechanical plough grooves and scratches. The coating flakes and delamination were also observed as a result of the brittle fatigue fractures caused by the continuous load (Figure 9b). The corresponding surface composition measurements on the worn surface are shown in Figure 9c,d. It can be considered that there existed little adhesive wear owing to the absence of iron element. Therefore, the main wear mechanisms of the Zr coating were abrasive wear, coating flaking, and delamination. The test results also identified that the tribological performance and property of the tested samples depended upon the materials of the friction pairs and the test conditions.

## 4. Conclusions

Pure Zr coating was deposited on the surface of a YT14 cemented carbide substrate using a multi-arc ion plating method. Dry sliding friction tests against a 40Cr hardened steel ring were implemented with the Zr-coated samples and traditional ones, and the main conclusions were obtained as below:

1. PVD Zr coating deposited on the carbide surface exhibited excellent adhesive strength. The coating adhesion strength reached about 60 N. The surface micro-hardness of Zr coating was about 12 GPa, the coating thickness was about 3.0 µm, and the surface roughness Ra was about 85 nm;
2. The average friction coefficient of Zr coated carbide was 20%–30% lower than that of the uncoated one under the same sliding test conditions. The coefficient of friction decreased with the increasing applied load, and changed slightly with varying sliding speed;
3. The Zr coated carbide could reduce the adhesive wear compared with the uncoated one, and the main wear mechanisms of the Zr coating were abrasive wear, coating flaking, and delamination.

## Figures and Tables

**Table 1:** Mechanical properties of the cemented carbide material.

Composition (wt. %)	Density (g/cm^3^)	Hardness (GPa)	Flexural Strength (MPa)	Young′s Modulus (GPa)	Thermal Expansion Coefficient (10^−6^/K)	Poisson′s Ratio
WC + 14%TiC + 6%Co	11.6	15.4	1200	510	6.50	0.25

**Table 2:** Deposition parameters of the Zr coating.

Substrate	Base Pressure (Pa)	Temperature (°C)	Ar Pressure (Pa)	Zr Current (A)	Deposition Temperature (°C)	Deposition Time (min)
Cemented carbide	6.5×10^−3^	220	0.6	90	250	90

**Table 3:** Element content analysis of worn surface for the Zr coating.

Element Content (Unit)	Point A in Figure 2a (wt. %)	Point B in Figure 2a (wt. %)
Zr	100	92.37
W		4.04
Ti		2.51
C		1.08
Total	100.00	100.00

**Table 4:** Mechanical properties of the Zr coating.

Substrate	Coating	Micro-Hardness (GPa)	Thickness (μm)	Adhesion Strength (N)	Surface Roughness Ra (nm)
Cemented carbide	Zr	12.0 ± 0.5	3.0 ± 0.1	60 ± 5	85 ± 5
